# KAN-Former: a lightweight ECG model for real-time atrial fibrillation detection on wearable devices

**DOI:** 10.3389/fbioe.2026.1824364

**Published:** 2026-05-28

**Authors:** Kaixuan Wang, Guozhen Wang, Zhiyong Wu

**Affiliations:** 1 School of Computer Science and Technology, Shandong University of Technology, Zibo, China; 2 Tumor Center, Yantai Affiliated Hospital of Binzhou Medical University, Yantai, China

**Keywords:** atrial fibrillation, ECG classification, lightweight neural networks, Kolmogorov-Arnold Networks (KAN), wearable devices

## Abstract

Atrial fibrillation (AF) is the most prevalent sustained cardiac arrhythmia and a major contributor to stroke and heart failure. Continuous monitoring using wearable electrocardiogram (ECG) devices enables early detection, but deploying deep learning models on resource-constrained platforms remains challenging due to limited computation and stringent real-time latency requirements. To address these challenges, we propose KAN-Former, a lightweight hybrid ECG classification model. KAN-Former consists of: (1) a multi-scale convolutional front-end enhanced with channel attention to capture morphological details across different temporal resolutions; (2) a Nyström-approximated Transformer module that models global temporal dependencies with linear complexity; (3) a time–frequency feed-forward network that refines spectro-temporal rhythm representations; and (4) a sparsity-optimized residual KAN classifier that improves representational efficiency and reduces parameter count. Experimental results on PhysioNet 2017 and CPSC 2018 demonstrate that KAN-Former achieves accuracies of 94.5% and 94.6%, respectively, with only 1.16M parameters and an inference latency of 3.8 ms, outperforming state-of-the-art lightweight models while maintaining real-time on-device performance. Furthermore, even under aggressive pruning and INT8 quantization, the accuracy degradation remains below 0.5%, confirming the scalability of KAN-Former for wearable AF screening.

## 1 Introduction

Atrial fibrillation (AF) is the most common sustained cardiac arrhythmia worldwide, affecting approximately 2%–3% of the general population, with its prevalence rising sharply to nearly 9% in individuals over 80 (Chousou et al., 2023). As a major risk factor for stroke, heart failure, and cardiovascular mortality, AF necessitates early, accurate, and automated detection (Kreimer et al., 2022). The electrocardiogram (ECG) remains the gold standard for AF diagnosis, but manual interpretation is labor-intensive and impractical for large-scale or long-term monitoring (Tonko and Wright, 2021; Martinek et al., 2022). Wearable and portable ECG devices now enable continuous cardiac monitoring, offering a practical pathway for real-time AF screening in daily life.

However, deploying accurate deep learning models on wearable systems faces significant challenges due to limited computational resources, constrained memory capacity, and strict real-time inference requirements (Park and Bae, 2024). Furthermore, ECG signals acquired from wearable sensors are often contaminated by baseline wander, motion artifacts, and muscle noise, which can compromise algorithm robustness (Basso et al., 2023). Current AF detection methods struggle to balance three key aspects: (1) effectively modeling multi-scale morphological and temporal dependencies in ECG signals (Chen et al., 2024); (2) capturing long-range temporal dependencies that encode crucial rhythm variability information (Basso et al., 2023); and (3) maintaining low computational cost and low latency for real-time on-device deployment (Wu et al., 2024).

The evolution of AF detection algorithms has progressed from traditional feature-based methods to modern deep learning approaches. Early techniques relied on handcrafted features such as irregular RR intervals or the absence of P-waves (Giraldo-Guzmán et al., 2021). With the rise of deep learning, convolutional neural networks (CNNs) and recurrent neural networks (RNNs) enabled end-to-end learning directly from raw ECG data, significantly improving diagnostic accuracy (Petmezas et al., 2022). For instance, Murat et al. provided a comprehensive review of deep learning approaches for AF detection, highlighting the superior performance of CNNs and long short-term memory networks (LSTMs) (Petmezas et al., 2022). More recently, hybrid architectures and Transformer-based models have been developed to better capture long-range dependencies. Haddi et al. demonstrated that combining univariate and multivariate data analysis could enhance AF detection accuracy (Haddi et al., 2022), while Wu et al. explored compressed CNNs specifically designed for wearable devices (Abdelazez, 2022).

Despite these advancements, most existing AF detection models remain difficult to deploy on resource-limited wearable devices because they either rely on computationally heavy architectures or are aggressively compressed at the expense of representational capacity (Murat et al., 2021). Lightweight CNN or RNN models often struggle to capture global temporal dynamics and long-range contextual interactions—both of which are crucial for stable AF recognition under noisy real-world conditions (Murat et al., 2021).

Recently, Kolmogorov-Arnold Networks (KANs) have emerged as a promising alternative to traditional neural networks (Wang et al., 2024). Unlike multilayer perceptrons (MLPs) or Transformers that rely on fixed linear transformations, KANs replace weight matrices with learnable spline-based univariate functions, enabling significantly higher expressiveness per parameter and providing intrinsic interpretability (Wang et al., 2024). Recent studies have shown that KANs can outperform MLPs and even Transformer-based models in physics-informed modeling, time-series forecasting, and medical pattern analysis, while using fewer parameters and maintaining high accuracy (Vaca-Rubio et al., 2024). For example, Liu et al. introduced a residual-KAN architecture that reduces parameter count without compromising performance (Wang et al., 2024), and Wang et al. developed lightweight KAN modules incorporating channel attention for edge AI deployment (Huang et al., 2025). These advancements highlight KANs as an emerging paradigm for efficient neural modeling; however, their potential for real-time AF detection on resource-constrained wearable devices remains largely unexplored. Our work bridges this gap.

To address these limitations, we propose KAN-Former (Kolmogorov-Arnold Network Transformer), a hardware-aware and lightweight hybrid architecture that integrates multi-scale convolution, efficient global temporal attention, and a sparsity-optimized KAN classifier. Unlike existing KAN-based ECG models such as Lightweight KAN (Huang et al., 2024) (which employs isolated KAN backbones with limited temporal modeling) and Federated KAN (Zeleke and Bochicchio, 2024) (which prioritizes distributed learning over architectural efficiency), KAN-Former achieves unique structural and efficiency gains through a hybrid design that synergizes multi-scale convolution, Nyström-based efficient attention, and a sparsity-optimized residual KAN classifier. This integration enables global temporal dependency modeling with linear complexity, time-frequency rhythm refinement, and parameter-efficient nonlinear mapping—resulting in a 1.16M model that delivers 3.8 ms inference latency and accuracy degradation below 0.5% under aggressive quantization, outperforming pure KAN or pure Transformer counterparts in both on-device feasibility and diagnostic accuracy.

Our main contributions are summarized as follows:

1. A multi-scale CNN front-end with channel attention that employs parallel depthwise separable convolutions to efficiently extract morphological features across multiple temporal resolutions.
2. An efficient Transformer module that leverages a Nyström-based approximation to capture long-range temporal dependencies with linear complexity.
3. A sparsity-optimized residual KAN classifier that replaces conventional multilayer perceptrons, achieving higher representational capacity per parameter through learnable univariate functions and embedded structural sparsity.
4. A hardware–algorithm co-design strategy, where structured pruning and post-training quantization are integrated into model development, resulting in a compact model capable of real-time on-device inference.

The remainder of this paper is organized as follows: Section 2 describes the materials and methods, detailing the KAN-Former architecture. Section 3 presents the experimental results and discussion. Finally, Section 4 concludes the paper.

## 2 Materials and methods

This section outlines the data preprocessing workflow and the architecture and design principles of KAN-Former, a lightweight hybrid model specifically optimized for real-time AF detection on wearable devices.

### 2.1 Data preprocessing

**FIGURE 1:**
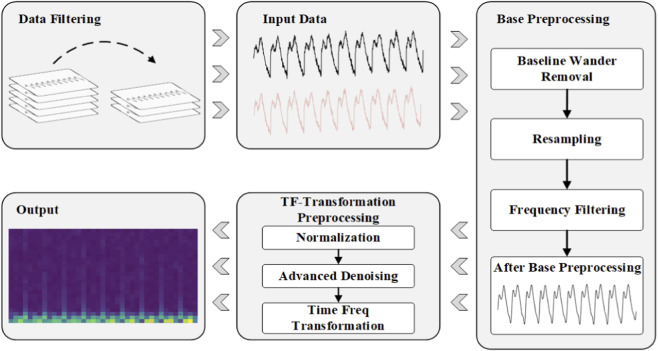
ECG data preprocessing pipeline.

Raw ECG signals were processed using a standardized six-step pipeline (Figure 1) that removed baseline drift, suppressed noise, normalized amplitude, and enhanced spectral patterns of arrhythmic events. This procedure ensured consistent input quality across datasets and device variations.

1. Baseline Wander Removal: A zero-phase fourth-order Butterworth high-pass filter (cutoff = 0.5 Hz) was applied to eliminate low-frequency drift due to respiration or electrode motion. Forward–backward filtering was used to avoid phase distortion (McCrae et al., 2022; Cos et al., 2023).
2. Frequency Filtering: A fourth-order Butterworth band-pass filter (0.5–40 Hz) was used to remove muscle artifacts and power-line interference while preserving essential morphological components for AF diagnosis (Wang et al., 2023; Andayeshgar et al., 2022).
3. Resampling: All ECG recordings were resampled to 200 Hz using a polyphase resampling approach, ensuring uniform temporal resolution and reducing computational load (Haque et al., 2022).
4. Amplitude Normalization: Each ECG segment was normalized to zero mean and unit variance (z-score) to minimize inter-patient amplitude variations and stabilize network training (Dambal et al., 2023).
5. Advanced Denoising: Motion-contaminated segments were denoised using Daubechies-4 wavelet decomposition (8 levels) during data augmentation, improving robustness against motion artifacts (Nahar, 2020).
6. Time–Frequency Transformation: The denoised 1D ECG signal was converted into a 2D spectrogram using the Short-Time Fourier Transform (STFT) to capture rhythm-dependent spectral characteristics. A 256-sample Hamming window with 75% overlap (hop size = 64) was used (Lazzaroni et al., 2021). The STFT is formulated as Equation 1:

$$S\left(m,k\right)=\sum_{n=0}^{N-1}x\left[n+mH\right]\cdot w\left[n\right]\cdot e^{-j2\pi fn/N}$$ (1)

where $x\left(n\right)$ is the preprocessed ECG signal at discrete time index n , $w\left[n\right]$ is the Hamming window of length N (set to 256 samples), H is the hop size (64 samples, corresponding to 75% overlap); m is the frame index; and k is the frequency bin index $\left(0\leq k\leq N-1\right)$ . The resulting spectrogram captures joint time–frequency dynamics essential for AF analysis. This spectrogram serves as the direct input to the KAN-Former model, replacing the raw 1D waveform for subsequent feature extraction.

### 2.2 KAN-former architecture

**FIGURE 2:**
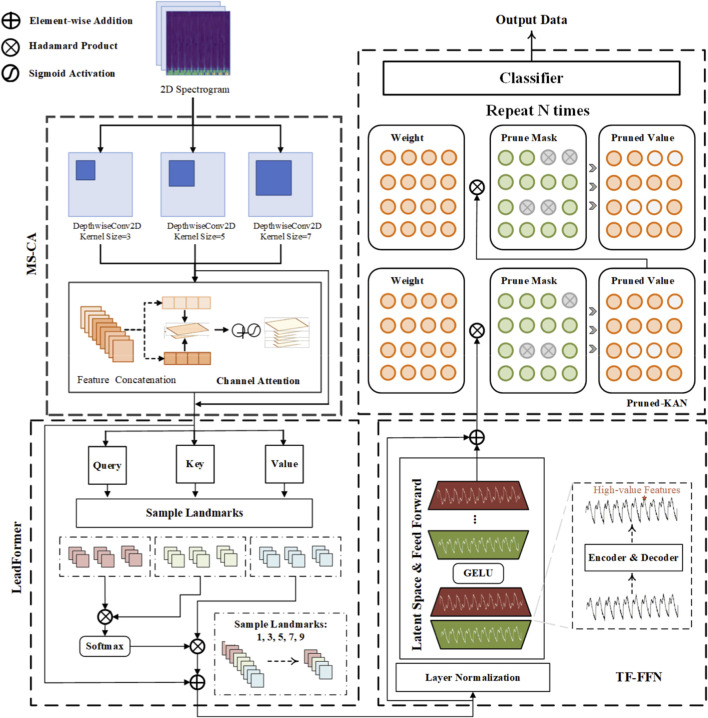
Overall architecture of KAN-former.

KAN-Former is a unified architecture designed for real-time AF detection on resource-limited wearable devices. As illustrated in Figure 2, the framework comprises four core components: (1) a Multi-Scale Convolution with Channel Attention (MS-CA) front-end for morphological feature extraction; (2) an efficient attention module (LeadFormer) with linear-complexity global temporal dependency modeling; (3) a Time–Frequency Feed-Forward Network (TF-FFN) for rhythmic pattern refinement; and (4) a sparsity-optimized Residual KAN classifier for compact nonlinear mapping. Each is designed to address specific challenges in ECG analysis while maintaining computational efficiency.

The overall transformation can be formally expressed as Equation 2:

$$y=Classifier\left(KAN\left(Projection\left(TF-FFN\left(LeadFormer\left(MS-CA\left(S\right)\right)\right)\right)\right)\right)$$ (2)

This hierarchical design enables progressive abstraction of ECG features—from time-frequency spectrogram patterns to high-level diagnostic representations—with each module contributing distinct analytical capability (Basso et al., 2023). The architecture follows hardware-aware design principles, emphasizing computational efficiency and minimal memory footprint to ensure deployability on wearable hardware.

### 2.3 MS-CA module

Cardiac electrical activity spans multiple temporal scales: fine-grained morphological cues (e.g., P-wave details) and broader rhythm patterns (e.g., R–R interval variability) both provide essential diagnostic information. Conventional approaches often struggle to concurrently capture these multi-scale features within computationally efficient architectures. The proposed MS-CA module addresses this challenge by combining parallel multi-scale convolutions and adaptive feature recalibration (Zhu et al., 2021).

The MS-CA module is designed around a multi-branch architecture that leverages 2D depthwise separable convolutions with varying kernel sizes of 3 × 3, 5 × 5, and 7 × 7 to process the input spectrogram. This allows the model to capture joint time–frequency patterns across different temporal and spectral resolutions. This design enables the extraction of complementary features across a hierarchy of temporal receptive fields. Specifically, the small kernel (size 3) captures fine-grained, local morphological details critical for identifying waveform boundaries, while the medium (size 5) and large (size 7) kernels progressively integrate broader contextual information, which is essential for analyzing rhythm stability (Liu J. et al., 2024). The use of depthwise separable convolutions significantly reduces computational complexity while retaining discriminative capacity (Yang et al., 2022).

The mathematical formulation of the multi-scale feature extraction is expressed as Equation 3:

$$F_{ms}=DWSConv_{k_{i}}\left(X\right),i\in \left\{1,2,3\right\}$$ (3)

where $X\in R^{C\times T}$ is the input ECG feature map (C channels, T time steps); $DWSConv_{k_{i}}$ denotes the depthwise separable convolution operation with kernel size $k_{i}$ (specifically $k_{1}=3,k_{2}=5,k_{3}=7$ ); and $F_{i}$ is the resulting multi-scale feature output. Depthwise separable convolution significantly reduces computational complexity compared to standard convolutions.

A lightweight channel-attention mechanism dynamically recalibrates multi-scale outputs as shown in Equation 4:

$$Attention=\sigma \left(W_{2}\cdot \delta \left(W_{1}\cdot GAP\left(F_{ms}\right)\right)\right)$$ (4)

where $W_{1}\in R^{\left(3C\times r\right)\times 3C}$ and $W_{2}\in R^{3C\times \left(3C\times r\right)}$ are learnable parameters, δ denotes the ReLU activation function, and σ represents the sigmoid function.

The final output is obtained through feature recalibration as given in Equation 5:

$$F_{out}=F_{ms}⊙Attention$$ (5)

where ⊙ denotes element-wise multiplication. This adaptive weighting allows the model to emphasize diagnostically relevant features while suppressing redundant information, enhancing the effectiveness of multi-scale representation (Rahman et al., 2024).

### 2.4 LeadFormer module

Although multi-lead ECG configurations provide rich spatial information, many wearable devices record only a single lead. To model the long-range temporal dependencies within a single-lead ECG signal efficiently, we propose the LeadFormer module. Conventional self-attention incurs quadratic complexity with sequence length, making it unsuitable for long ECG sequences on constrained hardware (Xiong et al., 2021). The LeadFormer module mitigates this limitation using a Nyström-based approximation that reduces computational complexity from $O\left(L^{2}\right)$ to $O\left(Lm\right)$ while preserving global contextual awareness (Xiong et al., 2021).

Given input features $X\in R^{B\times L\times C}$ from the MS-CA module, LeadFormer first projects them into query(Q), key (K), and value(V) representations using learnable linear transformations. The module employs multi-head attention with 4 heads to enable parallel processing across different representation subspaces, enhancing the model’s capacity to capture diverse aspects of long-range temporal dependencies within the sequence (Natarajan et al., 2021).

The core innovation lies in the landmark-based approximation strategy that strategically samples a subset of m positions $\left(m\ll L\right)$ to approximate the full attention matrix. Landmark selection employs uniform sampling with step size ⌊L/m⌋ , ensuring comprehensive coverage of the temporal sequence while maintaining computational efficiency as described in Equation 6:

$$landmark\_idx=arange\left(0,L,\lfloor L/m\rfloor \right)$$ (6)

The attention computation is then factorized using these landmarks as shown in Equation 7:

$$\begin{array}{l} Attention\left(Q,K,V\right)\approx Softmax\left(\frac{QK_{landmark}^{T}}{\sqrt{d_{k}}}\right)\cdot \\ Softmax{\left(\frac{Q_{landmark}K^{T}}{\sqrt{d_{k}}}\right)}^{T}\cdot V_{landmark} \end{array}$$ (7)

This approximation is mathematically grounded in the Nyström method for matrix completion and enables the module to capture long-range temporal dependencies that are essential for identifying rhythm patterns such as irregular R-R intervals. The module incorporates residual connections and layer normalization to ensure training stability and gradient flow, as expressed in Equation 8:

$$X_{out}=LayerNorm\left(X+Attention\left(Q,K,V\right)\right)$$ (8)

By reducing computational complexity while maintaining global receptive fields, LeadFormer effectively addresses a fundamental limitation of previous approaches that either sacrificed modeling capacity for efficiency or became computationally prohibitive for long ECG sequences typically encountered in continuous monitoring scenarios.

### 2.5 TF-FFN module

The non-stationary nature of ECG signals during arrhythmic episodes requires architectures capable of modeling both temporal dynamics and frequency-domain variability. The TF-FFN module addresses this challenge through a deep stack of gated feed-forward layers that progressively refine signal representations while maintaining computational efficiency suitable for on-device inference.

Each block in the TF-FFN implements a gated linear unit (GLU) transformation that enhances nonlinear modeling capacity compared to standard feed-forward networks (Qin et al., 2024). The transformation within each block is formally defined as Equation 9:

$$\begin{array}{c} Z=LayerNorm\left(X\right)\\ G=GELU\left(ZW1\right)\\ Output=Dropout\left(\left(G⊙\left(ZW2\right)\right)W3\right)+X \end{array}$$ (9)

where $W_{1}\in R^{C\times 2C}$ , $W_{2}\in R^{C\times 2C}$ and $W_{3}\in R^{2C\times C}$ are learnable parameters, and ⊙ denotes element-wise multiplication. The GELU activation function provides smooth nonlinear transformations that have demonstrated superior performance in deep networks compared to traditional ReLU activations (Dosovitskiy, 2020).

The two-layer depth of the TF-FFN enables progressive refinement of temporal representations: the first layer focuses on local morphological patterns, whereas the second layer integrates global context. The gating mechanism introduces a gating strategy that selectively propagates information based on the current context, effectively modeling the complex interactions between different frequency components in ECG signals.

Unlike attention mechanisms that introduce quadratic complexity, the TF-FFN’s feed-forward structure achieves deterministic, low-latency inference with linear complexity, making it well-suited for real-time wearable applications. The module incorporates dropout regularization (p = 0.1) between layers to prevent overfitting and improve generalization across heterogeneous patient data (Xiao et al., 2021).

### 2.6 KAN classifier

The final classification stage diverges from conventional MLP architectures by adopting KAN with structural sparsity and residual learning (Liu Z. et al., 2024). This approach is mathematically grounded in the Kolmogorov-Arnold representation theorem, which establishes that any multivariate continuous function can be represented as a finite composition of continuous functions of a single variable (Wang et al., 2024).

The fundamental building block of our implementation is the KANLinear layer, which replaces fixed activation functions with learnable univariate functions along the edges of the computational graph. Its forward process is defined as Equation 10:

$$y=\Phi \left(x\right)=\sum_{j=1}^{d_{in}}\phi_{ij}\left(x_{j}\right),with\;\phi_{ij}\left(x\right)=W_{base}\left[i,j\right]\cdot spline\left(x\right)\cdot G\left[i,j\right]\cdot M\left[i,j\right]$$ (10)

where $x\in R^{d_{in}}$ is the input vector, $y\in R^{d_{\text{out}}}$ is the output; $\phi_{ij}$ is a learnable univariate function associated with the edge from input node j to output node i ; $W_{base}\in R^{d_{out}\times d_{in}}$ is the base weight matrix; $\text{spline}\left(x\right)$ denotes a B-spline basis expansion; $G\in R^{d_{\text{out}}\times d_{\text{in}}}$ is a learnable gating matrix that modulates connection strengths; and $M\in {\left\{0,1\right\}}^{d_{out}\times d_{in}}$ is a binary pruning mask that enables dynamic sparsification during training (Bodner et al., 2024).

Our RKANLinear layer factorizes the weight matrix as W=A·B with $\in R^{\text{out}\times r},B\in R^{\text{in}\times r}$ , $r\leq \min\left(in,\text{out},64\right)$ .After training, we apply structured pruning (L2-norm, threshold λ = 0.1) to convolutional and linear layers, compute the mask once, then fine-tune. No pruning occurs during main training, ensuring full differentiability.

The full classifier stacks multiple KANLinear layers with residual connections and GELU activations, forming a compact yet expressive nonlinear mapping network. Residual connections mitigate gradient degradation, and KAN’s learnable univariate functions provide higher representational power per parameter, allowing the use of a compact dimension configuration (512, 128, 64) without sacrificing performance (Ji et al., 2024).

The KAN classifier offers three main advantages: (1) learnable univariate functions for ECG-specific feature representation (Pant et al., 2025); (2) structured sparsity for eliminating redundant parameters without accuracy loss (Chen and Ding, 2025); and (3) residual normalization for stable gradient flow and efficient training (Bodner et al., 2024).

### 2.7 Feature compression and classification pipeline

After feature extraction, a dedicated compression pipeline progressively reduces feature dimensionality while retaining discriminative temporal–spatial information essential for arrhythmia classification (Lu et al., 2021).

The compression pipeline begins with a depthwise separable convolution (kernel size = 3, padding = 1) that further processes the features while maintaining parameter efficiency through the factorization of spatial and channel-wise operations (Zhang et al., 2024). It is followed by a standard 1-D convolution that reduces channel dimensions by half, preserving salient activation patterns while lowering computational load (Huang et al., 2023). Adaptive max pooling reduces the temporal dimension to a fixed size of 16, retaining the most informative activation peaks.

A second convolution layer (kernel size = 3, padding = 1), followed by batch normalization and ReLU activation, performs further refinement. Global adaptive max pooling then generates a fixed-length feature vector. This progressive compression strategy maximizes retention of clinically meaningful ECG characteristics while eliminating redundancy and minimizing computation (Jin et al., 2023).

The compressed features are projected to the KAN input dimension via a linear layer with GELU activation and dropout (p = 0.1) and then processed by the sparsity-optimized KAN classifier with residual connections. The model outputs class probabilities through a linear classification head with enhanced dropout (p = 0.2), improving generalization across diverse patients and acquisition environments.

### 2.8 Hardware-aware optimization and deployment strategy

To bridge algorithmic performance with real-world deployment, computational complexity, memory usage, and power efficiency are jointly optimized. We adopt a hardware-aware optimization pipeline that integrates iterative structured pruning and post-training quantization (PTQ), ensuring that KAN-Former satisfies the latency and resource constraints of wearable bioelectronic systems without sacrificing diagnostic accuracy.

First, the model undergoes post-training structured pruning based on the L2-norm of convolutional filters and linear units. For each layer, we compute the L2-norm of each filter (or neuron), and remove those with norm below a global threshold λ (set to 0.1 after empirical tuning). The pruning ratio is not fixed but results from the threshold. After pruning, the remaining weights are fine-tuned for 10 epochs with a reduced learning rate (1e^−4^) to recover accuracy. No pruning occurs during the main training phase, so there is no need for gradient estimation through non-differentiable mask updates (Shen et al., 2024). This pruning is synergistic with the intrinsic sparsity of the KAN classifier, where the gating mechanism encourages sparse connectivity, further reducing computational overhead and improving cache-friendly memory access patterns suitable for edge accelerators.

After pruning, post-training quantization reduces weight and activation precision from 32-bit floating point (FP32) to 8-bit integer (INT8). This decreases the memory footprint by approximately 75% and enables execution on integer ALUs commonly available in edge AI processors. Quantization uses a symmetric calibration scheme on representative training samples, ensuring that the dynamic range of activations is preserved and avoiding saturation during inference (Zhou et al., 2024). The quantization scaling factor is computed as Equation 11:

$$\begin{array}{c} \text{scale}=\frac{\max\left(\left|W\right|\right)}{127}\\ W_{\mathrm{int}8}=\text{clip}\left(\text{round}\left(\frac{W_{\text{fp}32}}{\text{scale}}\right),-128,127\right) \end{array}$$ (11)

For mobile deployment, the trained PyTorch model is exported using joblib and converted to ONNX format, enabling inference on multiple execution backends (Shen et al., 2024). On Android, a dedicated mobile application is implemented in Android Studio (Java) and accelerated using ONNX Runtime for Mobile, enabling lightweight, dependency-free on-device inference. All processing stages—including preprocessing (filtering and STFT), feature extraction, and classification—are executed entirely on the device, eliminating reliance on external servers or cloud connectivity.

To further enhance real-time performance, time-critical components are implemented in C++ via Android NDK, while thread management, memory placement, and power consumption optimizations are applied at the OS level. These optimizations avoid garbage-collection stalls and improve data locality for continuous streaming inference.

As a result, the optimized KAN-Former achieves real-time inference (3.8 ms per ECG segment) with negligible accuracy loss (<0.5%), converting the model from a research prototype into a deployable, lightweight AF detection engine suitable for next-generation wearable bioelectronic monitoring systems.

## 3 Experimental results and discussion

### 3.1 Datasets

Two publicly available ECG datasets, PhysioNet/CinC 2017 and CPSC 2018, were employed to evaluate KAN-Former under diverse recording conditions. These datasets complement each other by covering both single-lead and multi-lead ECG scenarios, as summarized in Table 1:

1. PhysioNet/CinC 2017 Challenge Dataset: This dataset contains 8,528 single-lead ECG recordings (9–60 s, 300 Hz sampling rate) labeled into four classes: Normal, AF, Other Rhythm, and Noise. This is a widely used benchmark for AF detection (Dubatovka and Buhmann, 2022).
2. CPSC 2018 Dataset: A 12-lead dataset with 6,877 recordings (6–60 s, 500 Hz), annotated with nine diagnostic types. To evaluate AF detection in a more clinically realistic scenario where wearable devices may encounter various arrhythmias, we selected six clinically relevant classes: AF, I-AVB (first-degree atrioventricular block), LBBB (left bundle branch block), RBBB (right bundle branch block), PAC (premature atrial contraction), and PVC (premature ventricular contraction). This multi-class setup allows us to assess the model’s ability to distinguish AF from other common rhythm disturbances, which is critical for reducing false positives in real-world AF screening. To emulate single-lead wearable devices, only Lead II was used (Lai et al., 2021).

**TABLE 1:** Dataset specifications.

Dataset	Sampling rate	Records	Classes	Notes
PhysioNet2017	300 Hz	8,528	Normal, AF, Other Rhythm, Noise	Single-lead ECG
CPSC2018	500 Hz → 200 Hz	6,877	AF, I-AVB, LBBB, RBBB, PAC, PVC	Lead II extracted, multi-class

### 3.2 Experimental setup

Dataset Splitting: All datasets were stratified and split into training (70%), validation (15%), and test (15%) sets to maintain balanced class distributions.

All experiments were performed using PyTorch 2.3 on a workstation equipped with an Intel i7 CPU, 128 GB RAM, and an NVIDIA RTX 4090 GPU.

The model was trained for 50 epochs with a batch size of 64 using the AdamW optimizer (learning rate = 1 × 10^−3^, weight decay = 1 × 10^−4^) and the CosineAnnealingWarmRestarts scheduler. To mitigate class imbalance, a combination of weighted cross-entropy and focal loss (γ = 3) was applied. Data augmentation included Gaussian noise (σ = 0.01), amplitude scaling (±10%), and temporal shifting (±10%). All configurations are summarized in Table 2.

**TABLE 2:** Experimental setup for training and evaluation.

Category	Configuration/Model
Hardware	Intel i7 CPU, 128 GB RAM, NVIDIA RTX 4090 (24 GB)
Software	Python 3.12, PyTorch 2.3, CUDA 12.6
Dataset Split	Train 70%/Validation 15%/Test 15%
Batch Size	64
Epochs	50
Optimizer	AdamW (lr = 1e^−3^, weight decay 1e^−4^)
Scheduler	CosineAnnealingWarmRestarts
Loss Function	Weighted Cross-Entropy + Focal Loss

Post-training pruning: After the main training, structured pruning (λ = 0.1, L2-norm based) is applied to convolutional and linear layers, followed by 10 epochs of fine-tuning (lr = 1e^−4^). The KAN, classifier’s low-rank decomposition remains unchanged during pruning.

### 3.3 Evaluation metrics

Model performance was assessed using accuracy, F1-score, AUROC, parameter count, and inference latency, reflecting both diagnostic reliability and deployment efficiency:

- Accuracy—The proportion of correctly classified samples (Xu et al., 2022; Czmil, 2023).
- F1-score—Harmonic mean of precision and recall, effective for imbalanced datasets (Paragliola et al., 2023).
- Area Under the Receiver Operating Characteristic Curve (AUROC) — Measures the ability to discriminate among classes across thresholds (Roberts et al., 2024).
- Parameters (M) — Trainable parameter count, indicating model storage footprint (Azadbakht et al., 2022).
- Latency (ms) — Average inference time per ECG segment during testing (Dudziak et al., 2020).

This metric combination captures both diagnostic reliability and real-time efficiency—critical for wearable AI applications.

Multi-class evaluation for AF screening. Although the primary goal of this work is AF detection, we report results on the 6-class CPSC 2018 task for two reasons: (1) AF screening algorithms must reject non-AF arrhythmias to avoid unnecessary medical follow-up; (2) the 6-class setting provides a more rigorous benchmark for the model’s discriminative capacity. In all performance metrics (accuracy, F1-score, AUROC), we treat AF as the positive class while the other five classes are considered negative. The multi-class classification performance (e.g., F1-score of 0.824) reflects the model’s overall ability to correctly identify AF among other rhythm types, thereby supporting its practical utility in wearable AF screening.

### 3.4 Performance comparison on benchmark datasets

We benchmarked KAN-Former against a diverse set of representative models, including CNN-based architectures, lightweight Transformer variants, and recently proposed KAN-based frameworks. Experiments were conducted on the PhysioNet2017 and CPSC2018 datasets under identical preprocessing pipelines and dataset splits. For fair comparison, reported metrics from baseline models were extracted from their respective publications; parameter counts and inference latency marked with an asterisk (*) were estimated based on their architectural descriptions.

**FIGURE 3:**
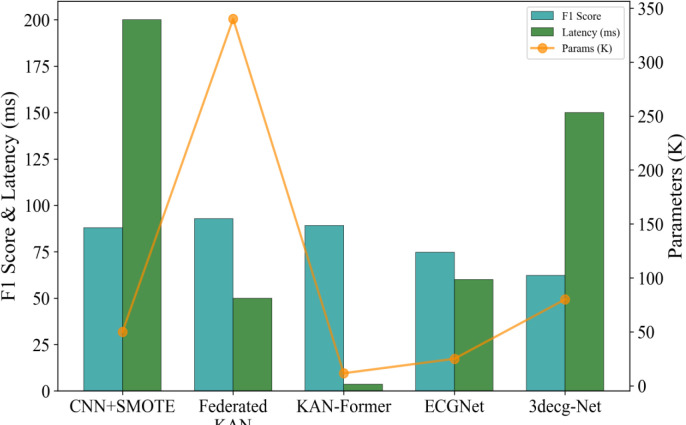
Performance-efficiency trade-off of different models.

Table 3; Figure 3 summarize and visualize the performance of KAN-Former compared to representative baseline models. This composite chart illustrates the critical trade-off between diagnostic performance (F1-score, shown as bars), operational efficiency (inference latency, also shown as bars, on the primary y-axis), and model complexity (parameters, represented by the line on the secondary y-axis).

**TABLE 3:** Comparison of KAN-Former and baseline models.

Model	Dataset	Accuracy	F1-score	AUROC	Params	Latency
CNN + SMOTE (Pandey and Janghel, 2019)	PhysioNet2017	0.979	0.880	–	∼5M*	200 ms
Tiny-Transformer (Busia et al., 2024)	PhysioNet2017	–	–	–	6 k	∼1 ms*
Federated KAN (Zeleke and Bochicchio, 2024)	PhysioNet2017	0.937	0.929	-	∼34M*	>50 ms*
KAN-Former (ours)	PhysioNet2017	0.945	0.891	0.947	1.16M	3.7 ms
ECGNet (Kim and Sunwoo, 2024)	CPSC2018	0.941	0.747	-	∼2.5M*	∼60 ms*
3decg-Net (Sadeghi et al., 2024)	CPSC2018	-	0.622	0.836	∼8M*	∼150 ms*
Lightweight KAN (Huang et al., 2024)	CPSC2018	-	-	0.948	∼1.5M*	∼15 ms*
KAN-Former (ours)	CPSC2018	0.946	0.824	0.932	1.16M	3.8 ms

The asterisk (*) indicates estimated values. Specifically, for the baseline models (CNN+SMOTE, Tiny-Transformer, Federated KAN, ECGNet, 3decg-Net, Lightweight KAN), the parameter counts and inference latency marked with * were not explicitly reported in their original publications. These values were estimated based on the architectural descriptions provided in those papers.

As clearly observed in Figure 3, the evaluated models exhibit distinct performance-efficiency characteristics. KAN-Former (ours) achieves a highly desirable profile, combining a high F1-score (0.891) with ultra-low latency (3.7 ms), while maintaining a compact model size (1.16M parameters), as indicated by its position on the parameter line. This optimal balance underscores its success in bridging the gap between clinical-grade accuracy and practical deployability.

When compared to high-accuracy models, the CNN + SMOTE model achieves a competitive F1-score of 0.880. However, its inference latency is prohibitively high (200 ms), and as the parameter line shows, its model size is approximately 4.3 times larger than that of KAN-Former. Such a computational footprint makes it unsuitable for deployment in resource-constrained environments. In contrast, KAN-Former delivers comparable accuracy with a reduction in latency by two orders of magnitude and a drastic reduction in model size.

Compared to other KAN-based approaches, Federated KAN achieves a slightly higher F1-score (0.929) but at a severe cost, requiring nearly 30 times more parameters and suffering from a latency over 13 times greater than KAN-Former. Conversely, while Lightweight KAN maintains efficiency with a comparable model size, it fails to match the diagnostic performance of our model due to its reliance on an isolated KAN backbone. This comparison underscores our architecture’s superior efficiency in leveraging the KAN paradigm. Where Federated KAN sacrifices practicality for performance and Lightweight KAN sacrifices accuracy for efficiency, KAN-Former strikes an optimal balance by moving beyond a simple classifier replacement to a deeply integrated, holistic design that synergizes KAN with specialized feature extractors for both high accuracy and real-time performance.

On the CPSC2018 dataset, KAN-Former’s advantage in balanced performance is evident. It significantly outperforms ECGNet (F1-score: 0.824 vs. 0.747) and 3decg-Net (F1-score: 0.824 vs. 0.622). Concurrently, Figure 3 shows that KAN-Former maintains significantly lower latency and a smaller parameter count than these baselines, confirming its superior speed and model efficiency.

### 3.5 Real-world wearable platform validation

To validate the deployability of KAN-Former on actual wearable hardware, we evaluated the optimized INT8 quantized model on an ARM Cortex-M4 microcontroller (STM32F407, 168 MHz, 192 KB SRAM, 1 MB Flash) representative of typical wearable processors. The model was converted to TensorFlow Lite for Microcontrollers and deployed with CMSIS-NN acceleration.

The detailed performance metrics on ARM Cortex-M4 are reported in Table 4. These results demonstrate that KAN-Former meets the strict resource constraints of wearable devices: inference latency is well below the typical real-time requirement (<100 ms), memory usage fits within the SRAM of low-power microcontrollers, and the energy per inference enables extended battery-operated monitoring. Compared to desktop GPU latency (3.8 ms, reported in Table 3), the 12.4 ms on Cortex-M4 remains fully adequate for real-time AF screening, while the quantization and pruning strategies reduce the model size from 1.16M parameters (FP32) to 298 KB (INT8), achieving a 74% reduction in memory footprint. This confirms that KAN-Former is not only algorithmically efficient but also practically deployable on resource-constrained wearable platforms.

**TABLE 4:** Real-world performance on ARM Cortex-M4.

Metric	Value
Inference latency per ECG segment (9 s)	12.4 ms
Peak RAM usage	86 KB
Flash memory footprint	298 KB
Energy per inference	0.63 mJ
Power consumption (continuous, 1 Hz inference rate)	0.63 mW

### 3.6 Ablation studies

To assess the contribution of each module, an ablation study was conducted on PhysioNet 2017. All ablation variants were trained using the exact same training settings (optimizer, learning rate schedule, batch size, loss function, data augmentation, and number of epochs) and the identical input preprocessing pipeline described in Section 2.1, ensuring a fair and reproducible comparison. The design of each ablated variant is formalized as follows:

1. Without Multi-scale: The MS-CA module is replaced by a single depthwise separable convolution layer with kernel size 3 and stride 1, keeping the same number of output channels as the original MS-CA module. This removes multi-scale feature extraction while maintaining a similar base convolutional capacity.
2. Without LeadFormer: The entire LeadFormer module (including its Nyström-based attention, residual connections, and layer normalization) is skipped entirely. The output features from the MS-CA module are directly fed into the TF-FFN module without any temporal dependency modeling.
3. Without TF-FFN: The TF-FFN module is removed. The output from LeadFormer is directly passed to the subsequent compression pipeline and then to the KAN classifier, bypassing the time-frequency refinement blocks.
4. Without KAN: The sparsity-optimized residual KAN classifier is replaced by a standard multi-layer perceptron (MLP) with a comparable parameter count (0.53M). The MLP consists of two linear layers (dimensions: 512 → 128 → 64) with GELU activation and dropout (p = 0.1), followed by a linear classification head. Residual connections and batch normalization are retained for fair comparison.

Results are presented in Table 5.

**TABLE 5:** Ablation study on PhysioNet2017.

Model variant	Accuracy	F1-score	AUROC	Params	Latency
Full KAN-Former	0.945	0.891	0.947	1.16M	3.7 ms
Without Multi-scale	0.816	0.733	0.834	0.86M	2.6 ms
Without LeadFormer	0.889	0.801	0.937	0.97M	2.8 ms
Without TF-FFN	0.893	0.871	0.939	0.93M	2.9 ms
Without KAN	0.905	0.825	0.948	0.53M	3.1 ms

**FIGURE 4:**
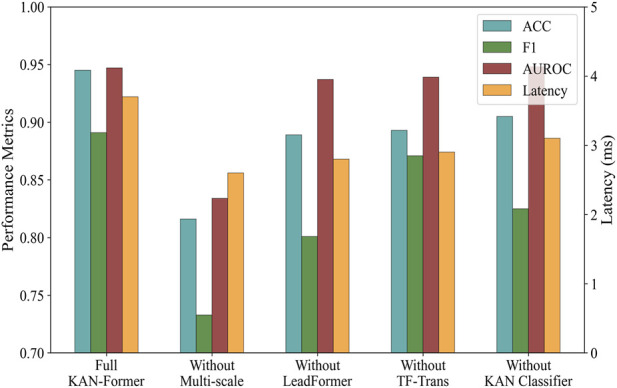
Ablation study results demonstrating the contribution of each component in KAN-Former.

To quantitatively assess the contribution of each core component, an ablation study was conducted, with the results detailed in Table 5 and visualized in Figure 4. The bar charts in Figure 4 provide a clear and immediate understanding of the performance impact caused by removing each module, offering critical insights into our architectural design:

1. MS-CA module is foundational: Removing the MS-CA block caused the most severe performance degradation, with accuracy dropping by 12.9% and F1-score by 15.8% (Figure 4). This empirically confirms that extracting morphological features across multiple temporal resolutions is crucial for accurate ECG analysis. The concomitant latency reduction (from 3.7 ms to 2.6 ms) indicates that the MS-CA module is a computational bottleneck. This finding justifies our use of efficient depthwise-separable convolutions within this module.
2. LeadFormer and TF-FFN offer Complementary Perspectives: The ablation of either the LeadFormer or the TF-FFN module led to a noticeable drop in F1-score (Figure 4), indicating that long-range temporal and time-frequency (rhythmic) features provide unique and complementary information for AF detection. The smaller performance penalty observed when removing LeadFormer on the single-lead PhysioNet2017 dataset is expected, and its importance is anticipated to be greater in longer sequences or multi-lead settings.
3. KAN Classifier enhances Representational Capacity Efficiently: Replacing the KAN classifier with a standard MLP, while reducing parameters and latency, consistently decreased accuracy and F1-score (e.g., −4.0% in F1-score, as seen in Figure 4). These findings suggest that the KAN provides a more efficient nonlinear mapping capability per parameter. Notably, the latency increase attributable to the KAN is minimal (∼0.6 ms), underscoring its significant value in enhancing model performance with a negligible computational overhead.

### 3.7 Visualization and decision insights

**FIGURE 5:**
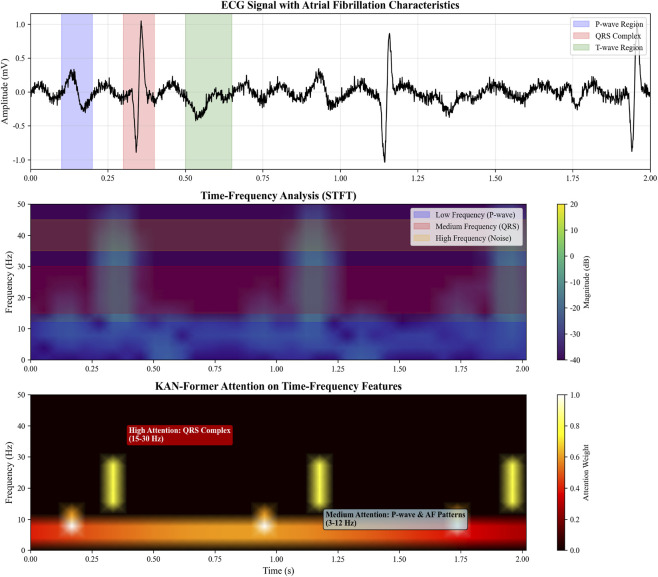
Visualization of time-frequency feature importance. The input ECG signal (top subplot) is transformed into a spectrogram (middle subplot). The overlayed heatmap (bottom subplot) represents the model’s feature importance scores. Red regions indicate high importance: **(1)** irregularly spaced high-frequency (10–30 Hz) peaks correspond to irregular R-R intervals; **(2)** absence of low-frequency (<5 Hz) activity before QRS complexes indicates missing P-waves—both are key clinical markers for AF. Blue regions indicate low importance. This alignment with standard cardiologist observations supports the physiological plausibility of KAN-Former.

To interpret model decisions and validate physiological alignment, feature importance maps from the TF-FFN module were visualized. This post-hoc analysis identifies time-frequency regions most influential in classification, as shown in Figure 5.

The visualization reveals that KAN-Former consistently focuses on physiologically meaningful ECG regions: (1) the medium-to-high frequency bands (10–30 Hz) during QRS complexes, corresponding to rapid ventricular depolarization; and (2) the low-frequency components (0.five to five Hz) preceding QRS complexes, representing P-waves associated with atrial activity. Notably, the model’s attention to P-wave absence or irregularity in low-frequency bands aligns with established clinical markers for AF diagnosis.

To further bridge model behavior with clinical criteria, we analyzed the temporal pattern of attended regions across consecutive beats. In AF samples, the model exhibits irregularly spaced high-importance peaks in the R-R interval range (frequency band around 5–10 Hz), directly reflecting the hallmark irregular R-R intervals of AF. Simultaneously, the persistent absence of significant low-frequency activity (<5 Hz) before QRS complexes corresponds to the absence of P-waves, another key clinical indicator of AF. In contrast, for normal sinus rhythm samples, the model consistently highlights regular low-frequency pre-QRS activity (P-waves) and evenly spaced QRS-associated high-frequency components. This direct mapping from model attention to clinical diagnostic rules enhances interpretability and supports the physiological plausibility of KAN-Former’s decision process.

These findings confirm that the model’s decision-making process correlates with clinically relevant ECG characteristics, enhancing trustworthiness for clinical integration.

## 4 Conclusion and future work

This paper has presented KAN-Former, a lightweight hybrid model for real-time atrial fibrillation detection on wearable devices. The proposed architecture combines multi-scale convolution, efficient global temporal attention, and a sparsity-optimized KAN classifier to achieve a superior accuracy-efficiency trade-off. Experimental results show that KAN-Former achieves high accuracy (94.5%–94.6%) with only 1.16M parameters and an inference latency of 3.8 ms, demonstrating strong deployment potential on resource-constrained devices. In addition, visualization results verify that the model focuses on clinically meaningful ECG regions, improving interpretability and trustworthiness.

Future work will explore validating the model on long-term ambulatory ECG data and extending its capability to multi-class arrhythmia diagnosis. Furthermore, optimizing KAN-Former for low-power microcontrollers and exploring patient-adaptive KAN kernels are promising directions for improving clinical applicability.

## Data Availability

The original contributions presented in the study are included in the article/supplementary material, further inquiries can be directed to the corresponding author.
